# Determinants of Femur and Tibia Fragility in Diabetes Using Dexamethasone-Induced Insulin Resistance in Rats

**DOI:** 10.1155/jdr/5781475

**Published:** 2025-10-28

**Authors:** Cheikh Ahmadou Bamba Mané, Maïmouna Touré, Cedric Wamba Koho, Herman Roger Sadie Foguieng, Khady Ngom, Hassane Malam Moussa Ahmet, Elvine Pami Nguelefack-Mbuyo, Abdoulaye Ba, Abdoulaye Samb, Télesphore Benoît Nguelefack

**Affiliations:** ^1^Laboratory of Physiology and Functional Explorations, Faculty of Medicine, Pharmacy and Dentistry, Cheikh Anta Diop University, Dakar, Senegal; ^2^Research Unit of Animal Physiology and Phytopharmacology, Faculty of Science, University of Dschang, Dschang, Cameroon; ^3^Laboratory of Pathological Anatomy, Faculty of Medicine, Pharmacy and Dentistry, Cheikh Anta Diop University, Dakar, Senegal

**Keywords:** bone fragility, dexamethasone, insulin resistance, oxidative stress

## Abstract

Type 2 diabetes, mainly driven by insulin resistance (IR), represents 90% of diabetic cases and has been related to bone fragility and fractures. However, it is unclear how hyperglycaemia, IR and oxidative stress interplay to induce bone fragility. This study was then undertaken to examine the contribution and the interconnection of hyperglycaemia, insulinopenia, IR and oxidative stress to bone fragility, as well as the time- and severity-dependent effects of insulin impairment on bone fragility and its reversibility. IR was induced by dexamethasone in three different protocols: 1 mg/kg/day/im for 7 consecutive days; 500 *μ*g/kg/day/im for 7 consecutive days, the dose was changed to 100 *μ*g/kg/day/im for the following 14 days; and 200 *μ*g/kg/day/im for 21 consecutive days. Fasting glycaemia, glucose tolerance test (GTT) and insulin tolerance test (ITT) were performed at the end of each treatment period while body mass was recorded daily. A control group receiving DMSO 4% was constituted for each protocol. Following euthanasia, the serum was collected for lipid profile, calcium, phosphate and alkaline phosphatase. The pancreas and liver were collected for the assessment of oxidative stress markers. Tibia and femur were collected to assess calcium phosphate and histological modifications. Dexamethasone induced an increase in baseline blood glucose and insulin intolerance while promoting glucose tolerance. A decrease in animal body weight as well as disturbances in oxidative stress, calcium and lipid profiles were observed in dexamethasone-treated animals. Bone histopathology showed loss of bone matrix, decreased collagen and an increase in Trap expression in dexamethasone-treated rats. Bone alterations were positively correlated with oxidative stress and IR. These results show that the phosphocalcic metabolism disruption and bone structure alterations induced by glucocorticoids are related to IR (time and severity) and oxidative stress status. Surprisingly, the relation is more effective on the tibia than the femur.

## 1. Introduction

Diabetes mellitus is a chronic metabolic disorder characterised by a permanent increase in blood glucose. It has become a global health concern with an augmenting prevalence worldwide [1]. Diabetes affects approximately 540 million individuals worldwide and, according to the International Diabetes Federation (IDF), approximately 783 million will be living with diabetes by 2045, an increase of 46% [2]. Diabetes mellitus significantly impacts various bodily systems, including bones. Indeed, bone fragility is a significant health concern that affects millions of people worldwide, particularly those with insulin resistance (IR) and diabetes.

Despite the increasing attention given to bone health and metabolic disorders in recent years, their intricate relationship remains not clearly understood. It is believed that IR is a hallmark of Type 2 diabetes (T2D). Conversely, individuals with T2D often exhibit normal or higher BMD [3], yet they still face a heightened risk of fractures due to alterations in bone microarchitecture and an environment that promotes osteoclast activity. It became, therefore, difficult to clearly define the role of carbohydrate metabolism in bone fragility, given that chronic T2D patients present moderate insulinopenia. Understanding the mechanisms underlying bone fragility in IR and diabetes is crucial for developing effective interventions to mitigate fracture risk in diabetic populations.

Other researchers have stipulated that the pathophysiological mechanisms accentuating fracture risk in T2D are convoluted, incorporating factors such as hyperglycaemia and insulinopenia [4, 5]. Chronic hyperglycaemia plays a pivotal role in this process, as it leads to the nonenzymatic glycation of collagen, compromising bone strength and integrity [6]. IR disrupts the delicate balance between bone formation and resorption. Recently, oxidative stress was suggested to contribute to bone fragility in diabetic patients [7]. But how IR, hyperglycaemia, insulinopenia and oxidative stress interplay to induce bone fragility remains to be elucidated.

This study was then undertaken to examine the contribution and the interconnection of IR, hyperglycaemia, insulinopenia and oxidative stress to bone fragility. Different approaches were used to characterise the time- and severity-dependent effects of insulin impairment on bone fragility, as well as its reversibility.

## 2. Materials and Methods

### 2.1. Reagents

Dexamethasone was obtained from Enzo Life Sciences (Switzerland). Thiobarbituric acid and urethane were obtained from Fluka (Germany). Trichloroacetic acid and hydrogen peroxide were purchased from Sigma-Aldrich (Germany), and orthophosphoric acid was purchased from BDH (Chemicals Ltd. Poole England). Insulin kits were obtained from Elabscience.

### 2.2. Animals

Animals used in this study were male and female *Wistar* rats aged between 3 and 4 months and weighing between 170 and 230 g. They were bred in colony cages under standard environmental conditions (temperature: 24°*C* ± 2°C, light/dark cycle: 12/12 h). The rats were fed with a standard commercial diet and water ad libitum. Animals were treated in accordance with the ethical guidelines for the use and care of laboratory animals as set out in European Parliament Law 2010/63/EU on the protection of animals used for scientific purposes and validated by the Faculty of Science Ethical Committee (Protocol 14-22/FS-CERFAS).

### 2.3. Experimental Protocol

**Figure 1:**
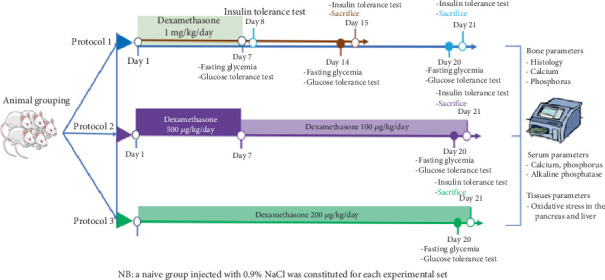
Study design for the evaluation of the effect of insulin resistance on bone health.

In order to assess the effect of the chronicity and the severity of IR on bone fragility and to examine whether short-term IR's negative impact on bone could be reversed, three different protocols were used. Animals were randomly assigned to different batches and groups. Baseline glycaemia was measured, and care was taken to ensure similar values between groups (Figure 1).

A solution of dexamethasone was prepared by solubilising 100 mg of dexamethasone in 3 mL of dimethyl sulfoxide (DMSO) contained in a graduated beaker. This solution was made up with 97 mL of 0.9% NaCl to give a final volume of 100 mL of dexamethasone. The dexamethasone solution was used as an IR inducer administered intramuscularly at a dose of 1 mg/kg at 100 *μ*L per 100 g body weight.

For the first protocol, IR was induced by intramuscular administration of dexamethasone at a dose of 1 mg/kg/day for 7 consecutive days in line with the modified protocol of Fofié et al. [8]. A total of 24 animals were divided into 02 batches (A and B), consisting of 02 groups each. Each group was made up of six rats (three males and three females) and treated as follows: Batch A consisted of Group 1 (naïve) that received 4% DMSO (1 mL/kg/day/im) and Group 2 (disease group) treated with dexamethasone (1 mg/kg/day/im); Batch B also consisted of Group 3 (naïve) that received 4% DMSO (1 mL/kg/day/im) and Group 4 (disease group) that received dexamethasone (1 mg/kg/day/im). Treatments in the two batches lasted for 1 week. The fasting glycaemia measurement and the glucose tolerance test (GTT) were performed on Day 7, while the insulin tolerance test (ITT) was carried out on Day 8. On Days 14 and 20, Batches A and B, respectively, underwent fasting blood glucose measurement and GTT, while ITT followed by euthanasia was performed on Day 15 (Batch A) and Day 21 (Batch B).

Protocol 2 was a modification of the one described by Nguelefack-Mbuyo et al. [9]. This new protocol was designed for sustained IR. Sixteen rats were divided into two groups of eight rats each (four males and four females) and treated as follows:

- Group 1 (naive) receiving 4% DMSO (1 mL/kg/day/im).
- Group 2 was injected intramuscularly with dexamethasone at the dose of 500 *μ*g/kg/day/im for 7 consecutive days. Then, the dose was changed to 100 *μ*g/kg/day/im for the following 14 days.

For Protocol 3, animals were divided and treated as in Protocol 2, except that the disease group received dexamethasone by intramuscular route at the dose of 200 *μ*g/kg/day/im for 21 consecutive days. This protocol shared the same control group with the second protocol. For Protocols 2 and 3, animals underwent the fasting glycaemia measurement and GTT on Day 20 and the ITT followed by euthanasia on Day 21.

For the three protocols, the different treatments were administered each morning between 7 and 9 AM. The body mass was recorded daily all along the experimental period. For euthanasia, animals were anaesthetised with thiopental (50 mg/kg) injected intraperitoneally. Thereafter, blood samples, pancreas, liver, tibia and femur were collected.

### 2.4. Oral GTT and ITT

The oral GTT was performed in animals fasted for 14 h. After the baseline blood glucose measurement, each animal was orally administered with 3 g/kg of glucose. Blood glucose was again measured at 30, 60, 90 and 120 min after glucose administration. Food was supplied immediately after the test.

Insulin solution (1 IU/mL) was prepared from a commercial stock solution of 100 IU/mL (Actrapid). To do this, 10 *μ*L of stock solution was diluted in 9.9 mL of 0.9% NaCl to obtain a final volume of 10 mL of 1 IU/mL insulin solution. This insulin solution was used for ITT. The ITT was performed the next day. After 6 h of fasting, the animal's baseline blood glucose level was measured, followed by subcutaneous injection of insulin (1 IU/kg). Then, the blood glucose level was determined at 30, 60, 90 and 120 min postinjection. Blood glucose was measured using a glucometer (Prodigy Diabetes Care, United States) with the tail blood.

### 2.5. Sample Preparation

The blood collected during euthanasia was centrifuged at 3500 rpm for 15 min. The serum obtained was stored at −20°C for subsequent analysis of insulin, calcium, phosphorus and alkaline phosphatase (ALP) levels. The liver and pancreas were homogenised at 15% in a Tris buffer (50 mM, pH = 7.4) and centrifuged at 10,000 rpm for 15 min at 4°C. The supernatant collected was aliquoted and stored at −20°C for the assay of biochemical markers such as malondialdehyde (MDA), superoxide dismutase (SOD), nitric oxide (NO), proteins and glutathione (GSH). Randomly, bones from the left foot were used for histological analyses while those from the right foot served to assay calcium and phosphorus. The latter were burned in an oven at 200°C, ground, suspended at 0.5g for 2 mL in demineralised water and then centrifuged at 3500 rpm. The supernatant was collected and used for calcium and phosphorus assays using the method described by Feki et al. [10]. The ratio 1/Ca∗P was also calculated and used in correlation analyses.

### 2.6. Biochemical Analysis

To assess the insulin levels, the ELISA commercial kit from Elabscience (Cat. # E-EL-R3034, Lot: drDQtY5TZL) was used. To evaluate the oxidant/antioxidant balance, MDA, GSH, SOD and NO were assayed as described by Fofié et al. [8]. Proteins were determined by the Biuret method.

### 2.7. Histological Analysis

The left femur and tibia from each animal were prepared for histological analysis. After fixation in 4% paraformaldehyde, the tibia and the femur were decalcified in 10% HCl for 7 days, dehydrated and embedded in paraffin. Sections of 5 *μ*m were stained with haematoxylin–eosin (HE) and Masson's trichrome. Digital microphotographs were obtained with a Leica DM 750 Camera.

For immunohistochemistry analysis of osteoclastic cells, 5 *μ*m paraffin-embedded sections of bone after decalcification were mounted on ionised superfrost slides. After dewaxing with xylene and hydrating with decreasing ethanol concentrations, the antigenic sites of the tissue section were repaired for 30 min in Tris-citrate buffer pH = 9. After washing with Tris-HCl buffer pH = 9, the sections were then immune-labelled using a polyclonal antibody TRAPPC6B (REF. PA5-113088; dilution 1:100; Invitrogen) according to the manufacturer's instructions.

### 2.8. Statistical Analysis of the Data

GraphPad Prism 8.4.2 software was used for the analyses. Results are expressed as mean ± SEM. Areas under the curve were automatically calculated. One-way analysis of variance (ANOVA) followed by Tukey's posttest was used to analyse one-variable parameters. In addition, two-way ANOVA followed by Bonferroni's posttest was used for bivariate data. Pearson's correlation was used to find the relationship between variables. *p* values were considered statistically significant when less than 0.05.

## 3. Results

### 3.1. Effect of Dexamethasone on the Body Mass in the Course of the Experiment

**Figure 2:**
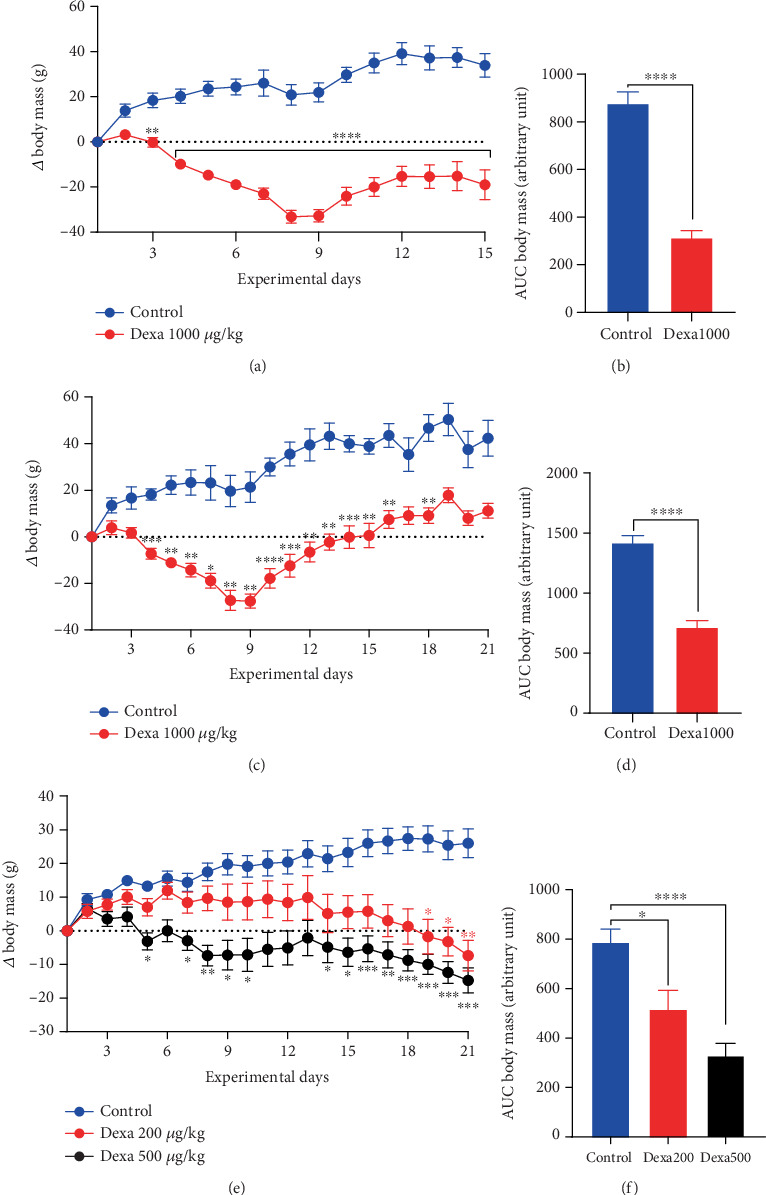
Dexamethasone treatment dose- and time-dependently reduced rat body mass. Each point or bar represents the mean ± SEM; *n* = 6–12. ⁣^∗^*p* < 0.05, ⁣^∗∗^*p* < 0.01, ⁣^∗∗∗^*p* < 0.001 and ⁣^∗∗∗∗^*p* < 0.0001 show significant difference using two-way ANOVA repeated measures with (a, c, e) Bonferroni's posttest and (b, d) *t*-test and (f) one-way ANOVA with Tukey's posttest.

As depicted in Figure 2a, dexamethasone administered at the dose of 1 mg/kg induced a significant reduction in animal body mass. The significant reduction started on Day 3 (*p* < 0.01) from the beginning of the administration. The maximal reduction (93%) was observed on Day 9 (*p* < 0.0001), 2 days after the cessation of the administration, and animals started recovering their body mass in a time-dependent manner. Dexamethasone-treated animals still showed significant (*p* < 0.01) low body mass on Day 18 as compared to control rats (Figure 2a,c). The overall analysis using the area under the curve exhibited a drastic drop (*p* < 0.0001) in dexamethasone-treated rats as compared to controls (Figure 2b,d).

It is noticed from Figure 2e,f that dexamethasone injected at doses of 200 and 500 *μ*g/kg/day induced a time- and dose-dependent reduction in body mass. Although a significant reduction compared to the control group was observed from Day 4 in animals receiving 500 *μ*g/kg/day and only from Day 18 in those treated with 200 *μ*g/kg/day (Figure 2e), the two treated groups did not show any significant difference between them (Figure 2f).

### 3.2. Effect of Dexamethasone on Basal Blood Glucose

**Figure 3:**
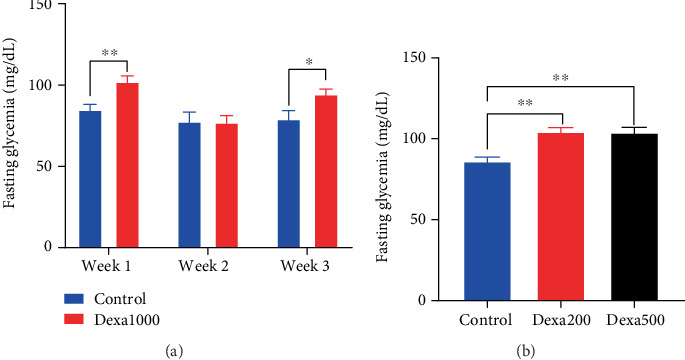
Dexamethasone induced time- and dose-dependent variation in fasting glycaemia. Each bar in the histogram represents the mean ± SEM; *n* = 6–12. ⁣^∗^*p* < 0.05 and ⁣^∗∗^*p* < 0.01 show significant difference using (a) multiple *t*-test or (b) one-way ANOVA with Tukey's posttest.

At the day before the start of the experiment, there was no significant variation in baseline blood glucose levels in rats receiving dexamethasone compared with controls. At the end of induction (end of Week 1) in rats receiving 1 mg/kg of dexamethasone, the baseline glycaemia was significantly higher (*p* < 0.01) than that of control rats. One week after the cessation of the treatment, no significant difference in baseline glycaemia was observed between the control and the dexamethasone-treated group. However, a significantly different result (*p* < 0.05) was seen between the two groups 2 weeks after the treatment was stopped (Figure 3a).

As shown in Figure 3b, dexamethasone administered at doses of 200 and 500 *μ*g/kg/day induced a dose-dependent increase (*p* < 0.01) in glycaemia as compared to the control group, but no significant difference was observed between the two dexamethasone-treated groups.

### 3.3. Effects of Dexamethasone on Glucose and Insulin Tolerance

**Figure 4:**
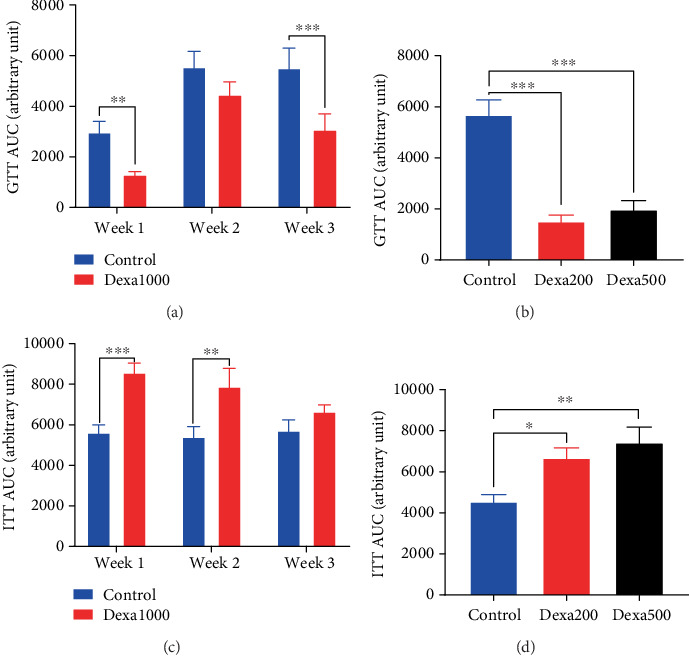
Intramuscular administration of dexamethasone impaired glucose and insulin tolerance. Each bar in the histogram represents the mean ± SEM. *n* = 6–12. ⁣^∗^*p* < 0.05, ⁣^∗∗^*p* < 0.01 and ⁣^∗∗∗^*p* < 0.001 show significant difference using (a, c) multiple *t*-test or (b, d) one-way ANOVA with Tukey's posttest.

The GTT and ITT were performed to assess both glucose homeostasis and insulin sensitivity. Results are expressed in Figure 4 as area under the curve. Animals treated with dexamethasone exhibited significantly (*p* < 0.01) better glucose tolerance than control. However, 1 week after the administration of 1 mg/kg, no significant difference was observed between control and disease animals, but a significant difference (*p* < 0.001) reappeared 2 weeks after the cessation of dexamethasone administration (Figure 4a). Animals treated for 21 days with dexamethasone (200 and 500 *μ*g/kg/day) exhibited significantly (*p* < 0.001) high tolerance to glucose (Figure 4b).

Concerning the insulin sensitivity test, 7 days' administration of 1 mg/kg/day of dexamethasone resulted in a reduction in insulin sensitivity, marked by a significant increase (*p* < 0.001) in area under the curve. One week after the cessation of the administration (Week 2), the insulin sensitivity was still significantly (*p* < 0.01) low in dexamethasone-treated animals, as compared to control, but the difference disappeared at Week 3 (Figure 4c). Dexamethasone at the doses of 200 *μ*g/kg/day (*p* < 0.05) and 500 *μ*g/kg/day (*p* < 0.01) also significantly impaired insulin sensitivity, but no difference was observed between the effect of the two doses (Figure 4d). Time-dependent variations in glycaemia during GTT and ITT are presented in the supporting information (Figure S1).

### 3.4. Effects of Dexamethasone on Insulinaemia

**Figure 5:**
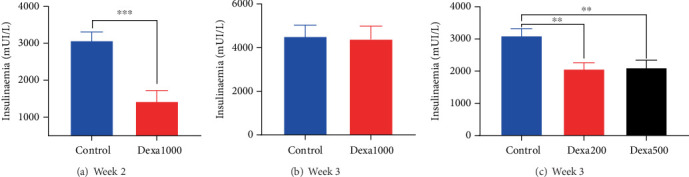
Dexamethasone intramuscular administration reduced blood insulin content. Each bar in the histogram represents the mean ± SEM; *n* = 6–8. ⁣^∗∗^*p* < 0.01 and ⁣^∗∗∗^*p* < 0.001 show significant difference using (a, b) *t*-test or (c) one-way ANOVA with Tukey's posttest.

As depicted in Figure 5, intramuscular administration of dexamethasone reduced the insulinaemia. In rats treated with the dose of 1 mg/kg, insulinaemia was significantly (*p* < 0.01) lower 1 week after the cessation of 7 days of consecutive administration of dexamethasone (Figure 5a) as compared to the control rat. At Week 3 (2 weeks after the end of treatment), dexamethasone-treated animals completely recovered, and their insulinaemia was similar to that of the control group (Figure 5b).

When dexamethasone was administered for 21 days, both protocols (200 *μ*g/kg/day or 500 *μ*g/kg/day + 100 *μ*g/kg/day) led to the significant (*p* < 0.01) reduction in blood insulin content compared to the control group (Figure 5c).

### 3.5. Variation of Homeostasis Model Assessment of Insulin Resistance (HOMA-IR) During the Experiment

**Figure 6:**
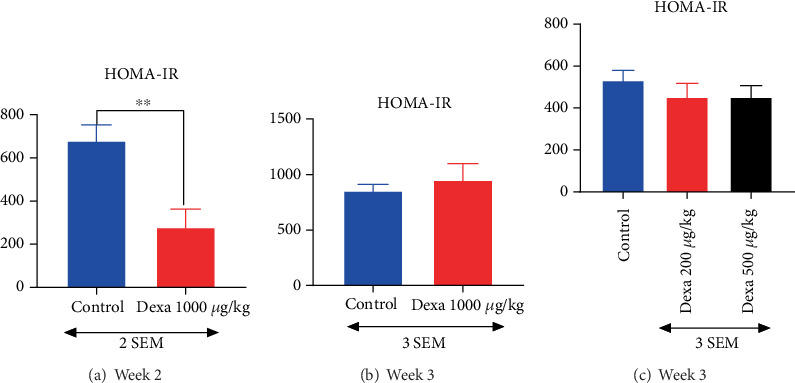
Intramuscular administration of dexamethasone induced variation of HOMA-IR according to the time and the dose. Each bar in the histogram represents the mean ± SEM; *n* = 6–8. ⁣^∗∗^*p* < 0.01 shows significant difference using (a) *t*-test.

The insulin level allowed us to calculate the HOMA-IR. In the first test, HOMA-IR was significantly (*p* < 0.01) lower 1 week after the cessation of 7 days consecutive administration of dexamethasone (Figure 6a) as compared to the control rat. However, in the group sacrificed at the end of Week 3, this trend was reversed, and IR was higher in the dexamethasone-treated rats (Figure 6b). In the second test, subsequent calculation of the HOMA-IR showed a nonsignificant decrease of HOMA-IR in the dexamethasone group compared with the controls (Figure 6c).

### 3.6. Effects of Dexamethasone on the Redox Status

Oxidative stress parameters were assessed in two target organs, the liver and pancreas, that have high impact on glucose homeostasis. Results are presented in Table 1. One week after the administration of dexamethasone at the concentration of 1 mg/kg/day, parameters such as GSH, SOD and NO did not show any significant variation in diseased animals, as compared to control. However, the liver MDA showed a significant decrease (*p* < 0.05) in dexamethasone-treated rats compared to controls. At Week 3, animals treated with 1 mg/kg dexamethasone had significant (*p* < 0.05) reduction in GSH and significant (*p* < 0.05) increase in NO in the pancreas. Rats treated with dexamethasone at the dose of 200 *μ*g/kg exhibited significantly (*p* < 0.05) low content of GSH in the liver and pancreas.

### 3.7. Effects of Dexamethasone on Phosphocalcic Profile

Table 2 presents the results of the calcium and phosphorus contained in the femur, tibia and serum, as well as the ALP level in serum. Dexamethasone induced a significant decrease in femur and serum calcium concentrations (*p* < 0.05). In the tibia, the parameter is significantly increased (*p* < 0.01). The same tendency was observed at 3 weeks in animals that received dexamethasone at 1000 *μ*g/kg/day, but only serum calcium concentration was significantly (*p* < 0.05) low compared to control. In rats that received dexamethasone at 500 *μ*g/kg/day, calcium was significantly (*p* < 0.05) low in femur, tibia and serum compared to control.

Concerning phosphorus, it was found significantly reduced only at 3 weeks in the femur of animals treated with 1 mg/kg/day (*p* < 0.01) and 500 *μ*g/kg/day (*p* < 0.05). In the serum, phosphorus was significantly (*p* < 0.05) increased in animals treated with 1 mg/kg/day while reduced in animals receiving 200 and 500 *μ*g/kg/day at the third week of the experiment.

The serum ALP was significantly (*p* < 0.05) increased at the end of Week 2 in rats that received 1 mg/kg/day compared to control. The same pattern was observed at Week 3 in all diseased animals.

### 3.8. Effect of Glycaemia Parameters and Oxidative Stress on the Phosphocalcic Balance

Correlation analyses presented in Table 3 show that the phosphocalcic ratio (1/Ca∗P) in the femur was not correlated to ITT, fasting glycaemia and oxidative index (OI). The tibial phosphocalcic ratio was correlated with the OI in the liver in rats treated with dexamethasone 200 *μ*g/kg/day (*r* = 0.88, *p* = 0.003).

The ALP serum was positively correlated with fasting glycaemia in the group receiving dexamethasone 200 *μ*g/kg/day (*r* = 0.75, *p* = 0.04). It was also positively correlated with the liver OI in rats treated with 500 *μ*g/kg/day of dexamethasone (*r* = 0.78, *p* = 0.03).

### 3.9. Bone Histopathological Analysis

**Figure 7:**
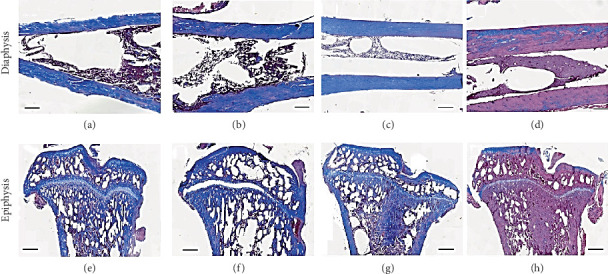
Histological section of diaphysis and epiphysis of tibia bone from control and dexamethasone-treated rats. (a, e) Control, (b, f) dexamethasone 200 *μ*g/kg, (c, g) dexamethasone 500 *μ*g/kg and (d, h) dexamethasone 1000 *μ*g/kg. Masson's trichrome staining.

**Figure 8:**
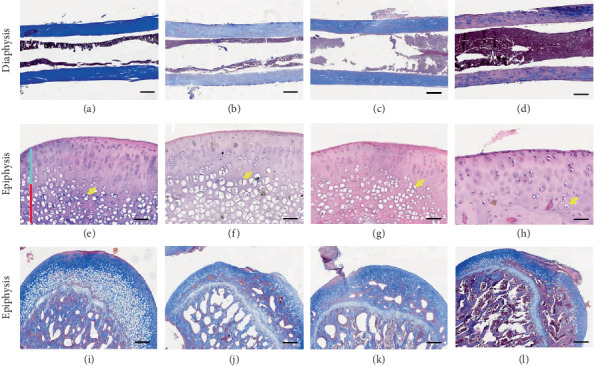
Histological section of diaphysis and epiphysis of femoral bone from control and dexamethasone-treated rats. (a, e and i) Control, (b, f and j) dexamethasone 200, (c, g and k) dexamethasone 500 *μ*g/kg and (d, h and l) dexamethasone 1000 *μ*g/kg. (e–h) are haematoxylin–eosin staining, and the rest are Masson's trichrome stained. The black scale represents 20 *μ*m. The blue light bar represents the superficial zone of the cartilage, and the red bar represents the transition zone. Yellow arrows indicate chondrocytes.

Results of the histological analysis of the tibia and femur are presented in (Figures 7 and 8), respectively.  Figure 7 shows the Masson trichrome staining with collagen expressed in blue.

In tibia bone, the collagen concentration was similar in the diaphysis (Figure 7a,b) and epiphysis (Figure 7e,f) of control and rats treated with dexamethasone 200 *μ*g/kg/day. The collagen content was slightly reduced in bone tissues of rats treated with the dose of 500 *μ*g/kg/day (Figure 7c,g) and drastically dropped in bone tissues of animals that received dexamethasone at the dose of 1000 *μ*g/kg/day (Figure 7d,h).

In the femur bone, the collagen content was reduced in both diaphysis (Figure 8a,d) and epiphysis of animals treated with the different doses of dexamethasone. In the epiphysis, the reduction seemed related to the dose of dexamethasone used (Figures 8i, 8j, 8k and 8l). Bones from animals treated with the dose of 1000 *μ*g/kg/day were more affected.

Figures 8e, 8f, 8g and 8h present epiphysis with HE staining, where chondrocytes can be observed in the transition zone. Compared to the control group (Figure 8e), the group treated with dexamethasone 200 *μ*g/kg/day (Figure 8f) exhibited high-density hypertrophied chondrocytes in the transition zone of the cartilage, while rats treated with 500 and 1000 *μ*g/kg/day (Figure 8g,h) showed a reduced number of chondrocytes, with the highest effect observed with the dose of 1000 *μ*g/kg/day.

**Figure 9:**
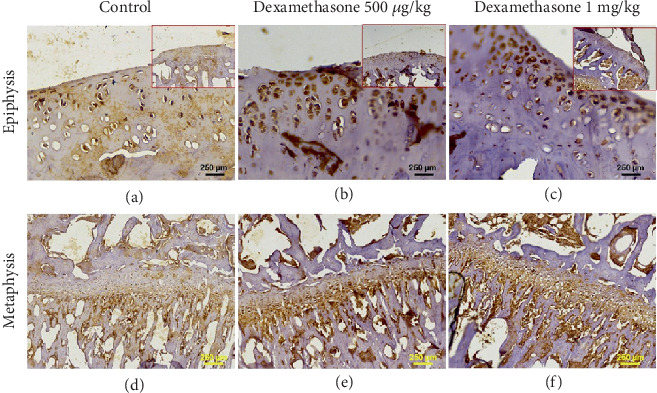
Immunohistochemistry of the (a–c) epiphysis and the (d–f) metaphysis of the tibia bone in dexamethasone-treated rat. Small frames at the top right of (a–c) represent the general view of the tissue at 100x. Dexamethasone treatment increased the expression of Trap in bone tissue. No significant difference in the Trap expression was observed between different doses.

In Figure 9, immunohistochemical analysis showed an increase in the expression of Trap in bone tissue in rats treated with dexamethasone. Figures 9a, 9b and 9c present the epiphysis, and Figures 9d, 9e and 9f present the metaphysis of the tibia bone in dexamethasone-treated rats. This trend was more marked in the tibia bone.

## 4. Discussion

IR is the starting point of T2D, resulting from a reduced capacity of target tissues to respond to circulating insulin concentrations. Several experimental models have been set up to reproduce the biological effects of IR in humans, notably the dexamethasone model [11, 12].

Beyond its cardiovascular complications, diabetes has deleterious effects on bone tissue. It is therefore an independent risk factor for bone fragility. The mechanism of bone fragility is not fully understood. Hence, the present study investigates the interaction between IR, oxidative stress and bone fragility in a dexamethasone-induced rat model of diabetes. Dexamethasone is widely used to induce IR in rodents. This model mimics several key features of T2D in humans, including reduced insulin sensitivity, hyperinsulinaemia, dyslipidaemia and cardiovascular disorders [13, 14].

The administration of dexamethasone at all doses resulted in a significant reduction in body weight, an increase in fasting blood glucose and glucose tolerance, a reduction in insulin sensitivity and insulinaemia and an increase in HOMA-IR, sprinkled oxidative stress, reduced bone phosphor–calcium and ALP content in the femur and serum, especially in rats treated with the dose of 500 *μ*g/kg/day. Bone collagen content and the density of chondrocytes were also reduced, while Trap expression was increased in the epiphysis and metaphysis in dexamethasone-treated animals.

Our results show that the administration of the different doses of dexamethasone resulted in a significant loss of weight in the animals. These results are in line with the data in the literature [8, 9] which showed a rapid decline for the 1 mg/kg dose and a progressive decline for the other doses in chronic treatment. These results show that the higher the dose of dexamethasone, the greater the loss of body mass. The same trend was observed in the work of other authors [9, 15] who showed that the drop in animal weight was due to metabolic changes such as hyperleptinaemia, which was responsible for the drop in food consumption. This reduction in lean mass is reflected in the muscle atrophy observed. In addition, authors have shown that dexamethasone induces muscle proteolysis leading to muscle atrophy [16, 17].

In the present study, it was observed that dexamethasone at all doses triggered hyperglycaemia, an increase in glucose tolerance and a reduced insulin sensitivity. These results corroborate those of other researchers [7, 14], and they have confirmed the presence of IR in rats treated with dexamethasone. Other studies have shown that dexamethasone causes hyperglycaemia by reducing glucose utilisation and stimulating hepatic glucose production [18, 19]. In the first test, the results also showed greater IR at the end of induction. Then, at the end of the second week, there was a drop in IR, followed by a significant resumption of IR at the end of the third week.

The decrease in insulin tolerance at the end of Week 2 can be explained by the hormetic effect. The hormetic effect is a tendency for pancreatic beta cells to correct glucose homeostasis to reestablish glycaemic balance as a result of the cessation of induction. At the end of Week 3, we noticed an increase in IR due to the exhaustion of compensatory mechanisms. This trend is in line with the work of Kolb and Eizirik [20] who assert the existence of the hormetic effect in T2D. Also, the variation in baseline glycaemia in our study concords with the results of other authors [21]. The drop obtained at the end of Week 2 brings out the U-shaped dose–response obtained. This finding has been reported in the literature by other studies [22].

In the second test, the different doses of dexamethasone produced significant IR compared to the controls. Following ITT, this IR was more obvious in the group receiving 500 *μ*g dexamethasone. Other authors [9] hold that IR depends on the dose and duration of the administration of dexamethasone. This is evident in the present work.

In rats receiving dexamethasone 1 mg/kg/day, the evaluation of HOMA-IR showed a decrease in IR at the end of Week 2 followed by an increase in IR at the end of Week 3. HOMA-IR is considered to be a reliable method of measuring IR [23, 24]. On a positive note, the results of this work are in line with the work of Shittu et al. [25] who also found an increase in HOMA-IR in rats treated with dexamethasone 1 mg/kg/day compared to controls. However, in the second test, IR was more patent in the group given 200 *μ*g/kg/day dexamethasone. These results corroborate the work of [24, 25] who showed that 200 *μ*g/kg was the best dose to induce IR in chronic administration.

Furthermore, the drop in IR observed at the end of Week 2, followed by an increase in IR at the end of Week 3 (U-shaped dose-response), as in the case of glycaemia, may be explained by the hormesis response. This hormesis response tends to rebalance glycaemic homeostasis at Week 2 and the exhaustion of compensatory mechanisms at the end of Week 3, hence the variation in IR.

The results of our study showed an imbalance in redox status in animals on dexamethasone. The 1 mg/kg/day dose led to a drop in MDA at Week 2 and an increase in NO at Week 3. These results contradict the views of researchers who illustrate an increase in MDA levels in animals treated with dexamethasone [9, 26, 27]. These results can be understood in the sense that the drop in MDA levels contrasts with an increase in NO levels. This is a compensatory mechanism that tends to restore redox balance in the organs [28]. However, for the second test, the results showed a significant drop in GSH and pancreatic SOD levels in the group receiving 200 *μ*g/kg/day. The oxidant parameter analysis showed a significant drop in liver MDA levels in the treated animals. The results of Test 2 support the view that through the reaction catalysed by GSH peroxidase reducing radicals by lowering levels of lipid peroxidation, GSH reduces hydrogen peroxide [29]. In addition, De Mattia et al. [30] showed that an infusion of reduced GSH improved insulin sensitivity. As a result, a drop in GSH and SOD can maintain IR. Also, in addition to the fall in antioxidant parameters, a fall in MDA levels has also been noted. This reflects lipid peroxidation, which, according to the literature review, should normally increase [9, 27] However, it is possible that the hormesis effect tends to balance the redox status in the organs.

IR results from a reduction in the ability of target tissues to respond correctly to normal circulating insulin concentrations [31]. Impaired insulin signalling may also have direct effects on bone metabolism, although the exact mechanism remains incompletely understood. Bone mineral composition and ALP are essential determinants of bone quality [32]. In our work, we noted a significant increase in ALP in insulin-resistant rats at all doses compared with controls. As demonstrated in the research conducted by Barlet et al. [33] who showed an increase in ALP in animal models of postmenopausal osteoporosis, an increase in ALP may be a useful indicator of osteoporosis. ALP, a marker of osteoblast differentiation and collagen synthesis, is a marker of osteoblast function [34]. In addition to the increase in ALPs, a decrease in calcium and phosphorus in the femur was noted in insulin-resistant animals. In fact, the increase in ALP associated with the decrease in phosphocalcic constituents of the bone is characteristic of osteoporosis [35].

Our results showed a drop in blood calcium levels in insulin-resistant animals. In the context of phosphocalcic regulation, hypocalcaemia is normally compensated by hypersecretion of parathyroid hormone; this secondary hyperparathyroidism leads to hyperresorption of bone [36], hence the increase in ALPs to promote bone remodelling, and this lack of compensation is at the root of osteoporosis.

Furthermore, glucocorticoids in pharmacological doses reduce intestinal calcium absorption, increase renal calcium excretion and counteract the effect of vitamin D [37]. Our results are contrary to the work of Pavel et al. [38] who found that the majority of osteoporosis patients had normal blood calcium levels. However, the increase in calcium levels observed in the tibia can be explained by the fact that the femur bone is more affected by osteoporosis than other bones in the body due to its size, anatomical position, function, cortical composition and vascularisation [39].

This imbalance in the phosphocalcium balance characteristic of osteoporosis can be explained by the fact that glucocorticoids exert direct effects on the different bone cell lineages, with inhibition of bone formation being the main feature [40, 41]. It is clear that hyperglycaemia and IR are capable of altering bone structure and function and impairing the extracellular matrix [42].

A positive correlation was observed between the liver OI and the phosphocalcium balance ratio in the tibia at a specific dose of dexamethasone. The OI is a concept used in biology and medicine to assess the level of oxidative stress in the body. It measures the body's ability to neutralise these free radicals and maintain a balance between reactive oxygen species (ROS) and antioxidants. Our study found a positive correlation between the OI in the liver and the decrease in calcium and phosphorus in the tibia. Our results are in line with the literature: the review of Hamada et al. [43] showed that oxidative stress inhibits osteoblastic differentiation and induces damage and apoptosis. In the same review, it is shown that spontaneously diabetic Torii rats, an animal model of T2D, exhibit low-turnover osteopenia associated with increased oxidative stress, and that markers of oxidative stress are inversely associated with histomorphometric parameters of bone formation.

Test results indicate that ALP is positively correlated with the OI in the pancreas of insulin-resistant rats. ALP is widely used in standard clinical practice; is present in serum and on the outer surface of most cells, mainly in the liver and bone; and plays an integral role in hepatobiliary and skeletal metabolism. Thus, serum ALP levels have long been considered a useful marker of hepatobiliary or bone disorders [44]. Our results show in the insulin-resistant rat group that ALP correlates positively with the OI in the pancreas. This same trend is reflected in the work of other authors, who showed that oxidative stress increases ALP activity in vascular, bone and intestinal epithelial cells [45, 46]. Torino et al. [47] reported that ALP activity correlates with whole-body biomarkers of oxidative stress in end-stage renal disease patients on dialysis.

In addition, our work showed that ALP was negatively correlated with baseline blood glucose levels in rats treated with 200 *μ*g/kg dexamethasone. In our work, our results showed a negative correlation between ALP and basal glycaemia; indeed, high blood glucose is capable of altering bone metabolism. This same trend was found in the work of other researchers [48, 49], who showed that high doses of glucose were capable of altering ALP activity and bone metabolism. In the group receiving a dose of 500 (100) *μ*g/kg dexamethasone, ALP was correlated negatively with calcium in situ in the femur. Our results showed a decrease in bone calcium in the femur, negatively correlated with ALP. Calcium and phosphorus, being minerals involved in bone consolidation, should evolve in the same direction as ALP. In contrast to our results, the work of Chen et al. [50] demonstrated that serum ALP levels were positively associated with Ca and P in these patients.

Analysis of microarchitecture and bone quality clearly showed that IR induced by dexamethasone caused massive and marked alterations in bone architecture and a reduction in diaphyseal thickness. This alteration was more marked for the 1 mg/kg/day dose of dexamethasone. Our results are in line with other authors [51, 52] who showed that the use of GCs resulted in an alteration of the bone, especially in the matrix, compared with the controls. In addition, our results showed a reduction in bone collagen in insulin-resistant rats. Collagen is one of the structures that gives bone its rigidity, so its reduction contributes to bone fragility and predisposes to pathological bone fractures. Our results corroborate the results of other authors who have shown that the use of glucocorticoids causes a reduction in collagen and predisposes to bone fragility [53, 54]. The HOMA index is a mathematical model for estimating IR. Our results are in line with those of [55], who found a positive correlation between HOMA-IR and the number of osteoclasts found in the group of ovariectomized rats (OVX-control) and the group of ovariectomized rats under treatment (OVX irisin-treated). He concludes that IR may be the cause of abnormal bone remodelling, leading to microstructural deterioration of the bone.

The strength of this study lies in the fact that many studies on IR do not assess insulinaemia and IR, which are important data for characterising IR induced by dexamethasone. This study also enabled us to characterise certain doses used in the literature and the duration of treatment, as well as the compensatory mechanisms that may occur with the use of these doses of dexamethasone. This study showed the impact of IR (the starting point for T2D) on bone tissue, and we were also able to assess the marker of bone formation (ALP), which was elevated in diabetic rats compared with controls. A possible explanation for the elevation in ALP could be related to an abnormal degree of mineralisation in diabetic bone. From this study, we can conclude that diabetic bone fragility could be related to a bone mineralisation defect by alteration of the “turn over” but also oxidative stress and lipid imbalance could be favouring factors.

On the other hand, the limitations of this study lie in the fact that histology of the liver and pancreas was not carried out, which would have enabled us to see the impact of the different doses morphologically. Evaluation of osteoblast activity by IHC would have provided a complete picture of bone remodelling. Assessment of insulin signaling pathways, particularly the PI3K pathway, would also have enabled us to understand the basis of IR induced by dexamethasone.

## 5. Conclusion

The main objective of which was to characterise dexamethasone-induced IR and the impact of this model on bone tissue, and the following conclusions can be drawn: (i) Dexamethasone at all doses increased fasting blood glucose levels. (ii) Tolerance tests showed an increase in glucose tolerance and IR in treated animals. (iii) The imbalance in the oxidant–antioxidant balance in favour of oxidative stress was present and variable depending on the dose and the duration of treatment. (iv) The study found a disturbance in the phosphocalcic balance, contrasting with an increase in ALP, which would seem to be linked to a lack of mineralisation. These factors, combined with latent oxidative stress and an imbalance in the lipid balance, may contribute to an increase in bone resorption and be at the root of bone fragility in diabetic bone.

Finally, it appears that IR can be induced by dexamethasone; in acute treatment, the dose of 1 mg/kg should be preferred and animals sacrificed immediately after induction to avoid compensatory mechanisms tending to reestablish organ homeostasis. These results will provide us with a model capable of mimicking T2D in humans, with a view to gaining a better understanding of the pathology and developing new therapeutics, as well as understanding the underlying causes of bone fragility in diabetic subjects.

## Figures and Tables

**Table 1:** Effects of dexamethasone on some oxidative and nitrosative stress parameters.

**Variables**	**Organs**	2 weeks		**3 weeks**		**3 weeks**		
		**Control** **n** = 6	**Dexa 1000** **n** = 6	**Control** **n** = 6	**Dexa 1000** **n** = 6	**Control** **n** = 8	**Dexa 200** **n** = 8	**Dexa 500** **n** = 8
GSH (*μ*mol/g tissue)	Hepatic	24.13 ± 3.37	30.29 ± 4.07	41.89 ± 4.79	38.98 ± 2.76	29.11 ± 2.73	22.07 ± 1.12^∗^	27.17 ± 2.40
	Pancreas	6.722 ± 0.67	6.204 ± 1.09	7.561 ± 0.74	5.112 ± 0.73^∗^	5.008 ± 1.15	2.225 ± 0.49^∗^	3.683 ± 0.68
SOD (Act mUI/g tissue)	Hepatic	228.8 ± 32.76	213.8 ± 25.08	145.0 ± 3.48	142.1 ± 12.32	156.8 ± 17.72	151.1 ± 14.60	153.5 ± 10.76
	Pancreas	366.0 ± 21.44	421.7 ± 40.60	393.9 ± 49.96	292.7 ± 37.31	327.1 ± 47.78	226.3 ± 24.39	385.3 ± 51.42
NO (*μ*mol/g tissue)	Hepatic	14.27 ± 3.66	18.09 ± 4.56	25.60 ± 2.87	26.42 ± 4.88	28.78 ± 2.51	30.87 ± 5.75	31.85 ± 4.55
	Pancreas	8.941 ± 2.81	6.552 ± 1.86	15.56 ± 2.08	25.74 ± 3.12^∗^	16.11 ± 4.37	11.67 ± 2.42	8.857 ± 2.62
MDA (*μ*mol/g tissue)	Hepatic	0.204 ± 0.04	0.091 ± 0.02^∗^	0.3127 ± 0.06	0.2327 ± 0.04	0.3675 ± 0.06	0.2758 ± 0.07	0.2888 ± 0.05
	Pancreas	0.1487 ± 0.03	0.1243 ± 0.02^∗^	0.08644 ± 0.02	0.0957 ± 0.02	0.1015 ± 0.01	0.1161 ± 0.01	0.06400 ± 0.01

*Note:* Each value represents the mean ± SEM. *N* = 6–8. Analysis is performed in the same line.

⁣^∗^*p* < 0.05, significant difference versus respective control using Student's *t*-test (Dexa 1000) or one-way ANOVA followed by Tukey's posttest.

**Table 2:** Effects of dexamethasone on phosphocalcic profile.

**Variables**		**2 weeks**		**3 weeks**		**3 weeks**		
		**Control** **n** = 6	**Dexa 1000** **n** = 6	**Control** **n** = 6	**Dexa1000** **n** = 6	**Control** **n** = 8	**Dexa 200** **n** = 8	**Dexa 500** **n** = 8
Calcium (mg/dL)	Femur	9.023 ± 0.41	5.830 ± 1.21⁣^∗^	10.28 ± 0.23	9.948 ± 0.35	10.1 ± 0.14	9.25 ± 0.84	8.21 ± 0.77^∗^
	Tibia	8.860 ± 0.33	10.20 ± 0.15^∗∗^	9.786 ± 0.42	10.68 ± 0.13	10.4 ± 0.22	8.51 ± 1.12	7.89 ± 0.83^∗^
	Serum	4.606 ± 0.18	3.991 ± 0.16^∗^	4.927 ± 0.20	4.166 ± 0.15^∗^	4.33 ± 0.05	4.33 ± 0.17	3.91 ± 0.19^∗^

Phosphorus (mg/dL)	Femur	1.189 ± 0.12	1.907 ± 0.41	2.638 ± 0.16	1.645 ± 0.22^∗∗^	1.900 ± 0.21	1.499 ± 0.23	1.323 ± 0.07^∗^
	Tibia	1.298 ± 0.18	1.352 ± 0.16	1.573 ± 0.26	1.623 ± 0.10	1.817 ± 0.12	1.682 ± 0.23	2.317 ± 0.35
	Serum	2.595 ± 0.37	2.305 ± 0.36	2.154 ± 0.20	3.492 ± 0.40^∗^	1.714 ± 0.17	1.191 ± 0.16^∗^	1.147 ± 0.16^∗^

ALP (UI/L)	Serum	20.96 ± 3.17	36.38 ± 5.40^∗^	32.17 ± 5.01	41.55 ± 4.10	21.93 ± 4.77	25.91 ± 7.62	23.00 ± 2.53

*Note:* Each value represents the mean ± SEM. Analysis was performed in the same line versus respective control using Student's *t*-test or one-way ANOVA followed by Tukey's posttest.

Abbreviation: ALP, alkaline phosphatase.

⁣^∗^*p* < 0.05 and ⁣^∗∗^*p* < 0.01, significant difference.

**Table 3:** Correlation between phosphocalcic profile (femur and tibia) and alkaline phosphatase with insulin resistance determinants.

		**Subchronic phase**		**Chronic phase**				
		**Control**	**Dexa 1000**	**Control**	**Dexa1000**	**Control**	**Dexa 200**	**Dexa 500**
Phosphocalcic balance (1/*Ca*∗*P*)—Femur	AUC ITT	*r* = −0.01	*r* = −0.22	*r* = 0.31	*r* = 0.32	*r* = 0.01	*r* = −0.12	*r* = 0.06
		*p* = 0.98	*p* = 0.67	*p* = 0.54	*p* = 0.53	*p* = 0.98	*p* = 0.78	*p* = 0.89
	Fasting glycaemia	*r* = −0.55	*r* = 0.35	*r* = −0.10	*r* = 0.54	*r* = −0.10	*r* = −0.19	*r* = −0.03
		*p* = 0.25	*p* = 0.55	*p* = 0.86	*p* = 0.20	*p* = 0.80	*p* = 0.67	*p* = 0.93
	IO—Liver	*r* = 0.58	*r* = −0.04	*r* = −0.11	*r* = 0.05	*r* = 0.42	*r* = 0.37	*r* = 0.13
		*p* = 0.22	*p* = 0.92	*p* = 0.83	*p* = 0.90	*p* = 0.28	*p* = 0.36	*p* = 0.75
	IO—Pancreas	*r* = 0.054	*r* = −0.44	*r* = −0.62	*r* = 0.07	*r* = 0.33	*r* = −0.49	*r* = −0.01
		*p* = 0.91	*p* = 0.32	*p* = 0.18	*p* = 0.86	*p* = 0.41	*p* = 0.21	*p* = 0.97

Phosphocalcic balance (1/*Ca*∗*P*)—Tibia	AUC ITT	*r* = 0.10	*r* = −0.45	*r* = −0.32	*r* = 0.32	*r* = −0.71	*r* = −0.45	*r* = 0.57
		*p* = 0.84	*p* = 0.36	*p* = 0.53	*p* = 0.53	*p* = 0.06	*p* = 0.30	*p* = 0.17
	Fasting glycaemia	*r* = 0.04	*r* = 0.14	*r* = 0.96	*r* = −0.12	*r* = −0.41	*r* = −0.71	*r* = 0.85
		*p* = 0.93	*p* = 0.81	*p* = 0.008	*p* = 0.79	*p* = 0.31	*p* = 0.07	*p* = 0.007
	OI—Liver	*r* = 0.08	*r* = 0.59	*r* = −0.22	*r* = −0.54	*r* = 0.15	**r** = 0.88	*r* = −0.39
		*p* = 0.86	*p* = 0.16	*p* = 0.67	*p* = 0.20	*p* = 0.72	**p** = 0.003	*p* = 0.33
	OI—Pancreas	*r* = −0.14	*r* = 0.04	*r* = −0.20	*r* = −0.003	*r* = 0.53	*r* = −0.51	*r* = −0.14
		*p* = 0.78	*p* = 0.92	*p* = 0.69	*p* = 0.99	*p* = 0.17	*p* = 0.19	*p* = 0.72

Alkaline phosphatase (UI/L)	AUC ITT	*r* = 0.12	*r* = 0.98	*r* = 0.20	*r* = 0.38	*r* = −0.22	*r* = 0.35	*r* = 0.32
		*p* = 0.81	*p* = 0.000	*p* = 0.69	*p* = 0.44	*p* = 0.62	*p* = 0.43	*p* = 0.47
	Fasting glycaemia	*r* = 0.60	*r* = 0.48	*r* = 0.49	*r* = 0.40	*r* = −0.33	**r** = 0.75	*r* = −0.05
		*p* = 0.20	*p* = 0.40	*p* = 0.39	*p* = 0.36	*p* = 0.42	**p** = 0.04	*p* = 0.91
	OI—Liver	*r* = 0.02	*r* = −0.39	*r* = −0.64	*r* = 0.24	*r* = −0.11	*r* = −0.38	**r** = 0.78
		*p* = 0.96	*p* = 0.44	*p* = 0.16	*p* = 0.60	*p* = 0.79	*p* = 0.34	**p** = 0.03
	OI—Pancreas	*r* = 0.55	*r* = 0.71	*r* = −0.28	*r* = 0.69	**r** = 0.69	*r* = −0.27	*r* = 0.000
		*p* = 0.25	*p* = 0.10	*p* = 0.58	*p* = 0.08	**p** = 0.05	*p* = 0.51	*p* = 0.99
	(1/*Ca*∗*P*)—Femur	*r* = −0.41	*r* = −0.20	*r* = −0.28	*r* = 0.40	*r* = −0.29	*r* = −0.32	*r* = 0.43
		*p* = 0.40	*p* = 0.69	*p* = 0.59	*p* = 0.36	*p* = 0.47	*p* = 0.43	*p* = 0.33
	(1/*Ca*∗*P*)—Tibia	*r* = −0.50	*r* = −0.52	*r* = 0.26	*r* = −0.19	*r* = 0.40	*r* = −0.16	*r* = −0.22
		*p* = 0.30	*p* = 0.28	*p* = 0.61	*p* = 0.67	*p* = 0.31	*p* = 0.69	*p* = 0.63

*Note:* Values marked in bold are considered significant. The phosphocalcic ratio (1/*Ca*∗*P*). *p* ≤ 0.05 is considered significant.

Abbreviation: OI, oxidative index.

## Data Availability

The data used to support the findings of this study are available from the corresponding author upon request.

## Supporting Information

Supporting Information Additional supporting information can be found online in the Supporting Information section. Figure S1: Time-dependent variations in glycaemia during glucose tolerance test (GTT) and insulin tolerance test (ITT).
