# Supplementary material for: Determinants of Femur and Tibia Fragility in Diabetes Using Dexamethasone-Induced Insulin Resistance in Rats
Source: J Diabetes Res. 2025 Oct 28;2025:5781475. doi: 10.1155/jdr/5781475 (PMC12585827; doi:10.1155/jdr/5781475)
Supplement: Supporting Information — Additional supporting information can be found online in the Supporting Information section. Figure S1: Time-dependent variations in glycaemia during glucose tolerance test (GTT) and insulin tolerance test (ITT). [file 5781475.f1.docx]

**Figure S1** : Time-dependent variations in glycemia during Glucose Tolerence Test (GTT) and Insulin Tolerance Test (ITT). Each point or bar represents the mean ± SEM ; n = 6 to 12. *p ˂ 0.05, **p ˂ 0.01, ***p ˂ 0.001, ****p ˂ 0.0001 significant difference using ANOVA two ways repeated measures with Bonferroni post-test. Panels (a, b, c, d, e, f) represents GTT and ITT with 1mg/kg/day at the end of week 1, week2 and week3. Panels (g and h) represents GTT and ITT with 200 µg/kg/day and 500[100] µg/kg/day at the end of the experiment (Week3).
